# A review on boiling heat transfer enhancement with nanofluids

**DOI:** 10.1186/1556-276X-6-280

**Published:** 2011-04-04

**Authors:** Jacqueline Barber, David Brutin, Lounes Tadrist

**Affiliations:** 1 Aix-Marseille Université (UI, UII)-CNRS Laboratoire IUSTI, UMR 6595, 5 Rue Enrico Fermi, Marseille, 13453, France

## Abstract

There has been increasing interest of late in nanofluid boiling and its use in heat transfer enhancement. This article covers recent advances in the last decade by researchers in both pool boiling and convective boiling applications, with nanofluids as the working fluid. The available data in the literature is reviewed in terms of enhancements, and degradations in the nucleate boiling heat transfer and critical heat flux. Conflicting data have been presented in the literature on the effect that nanofluids have on the boiling heat-transfer coefficient; however, almost all researchers have noted an enhancement in the critical heat flux during nanofluid boiling. Several researchers have observed nanoparticle deposition at the heater surface, which they have related back to the critical heat flux enhancement.

## Introduction

Boiling heat transfer is used in a variety of industrial processes and applications, such as refrigeration, power generation, heat exchangers, cooling of high-power electronics components and cooling of nuclear reactors. Enhancements in boiling heat transfer processes are vital, and could make these typical industrial applications, previously listed, more energy efficient. The intensification of heat-transfer processes and the reduction of energy losses are hence important tasks, particularly with regard to the prevailing energy crisis.

In terms of boiling regimes, nucleate boiling is an efficient heat-transfer mechanism; however, for the incorporation of nucleate boiling in most practical applications, it is imperative that the critical heat flux (CHF) is not exceeded. CHF phenomenon is the thermal limit during a heat-transfer phase change; at the CHF point the heat transfer is maximised, followed by a drastic degradation after the CHF point. Basically, the boiling process changes from efficient nucleate boiling to lesser-efficient film boiling at the CHF point. The occurrence of CHF is accompanied by localised overheating at the heated surface, and a decrease in the heat-transfer rate. An increase in the CHF of the boiling system would therefore allow for more compact and effective cooling systems for nuclear reactors, air-conditioning units, etc. For decades, researchers have been trying to develop more efficient heat-transfer fluids, and also to increase the CHF of the boiling system which would, in turn, improve process efficiency and reduce operational costs. This is where nanofluids could play a key role; nanofluids could potentially revolutionise heat transfer.

Nanofluids are colloidal suspensions of nanoparticles (length scales 1-100 nm) in a base fluid. These particles can be metallic (Cu, Au) or metal oxides (Al_2_O_3_, TiO_2_, ZrO_2_), carbon (diamond, nanotubes), glass or another material, with the base fluid being a typical heat-transfer fluid, such as water, light oils, ethylene glycol (radiator fluid) or a refrigerant. The base fluids alone have rather low thermal conductivities. Suspending particles in a base liquid to improve the thermal conductivity is not a new idea; previously the set back for scientists was the particle size. Manufacturing limitations in the past allowed only the creation of microparticles, and these particles quickly settled out of the fluid, and deposited in pipes or tanks, clogging flow passages, causing damage and erosion to pumps and valves, and increasing pressure drop. Nanoparticles, however, can be dispersed in base fluids and remain suspended in the fluid to a much greater extent than was previously achieved with microparticles. This is mainly thought to be due to Brownian motion preventing gravity settling and agglomeration of particles, resulting in a much more stable, suspended fluid.

Choi [1] first used the term 'nanofluids' in 1995, where he provided results of a theoretical study of suspended copper nanoparticles in a base fluid; he indicated abnormal improved thermal properties of the nanofluids. Further experimental investigations have reported that suspensions containing nanoparticles have substantially higher thermal conductivities than those of the base heat-transfer fluids [1-3]. This was initially considered abnormal since such a large enhancement in the CHF, as large as 200% in some cases [4], could not be interpreted through the existing CHF theories and models. What is also exciting is that only very small volume fractions, i.e. <1%, are required to show enhancement of the thermal base fluid.

Already, there has been significant research into the enhancements in nucleate boiling CHF by the use of nanofluids for pool boiling applications. Research on enhancements of CHF using nanofluids under convective flow conditions have been investigated, but to a lesser extent. It is also interesting to note that the majority of the experimental data provided in the literature are for enhancement effects of nanoparticles or nanofluids on the CHF condition. There is a significant gap in the data presented of the enhancement, which nanofluids have on the boiling heat transfer (BHT) coefficient, which is also a vital piece of information to know for their incorporation in heat-transfer applications. The BHT coefficient is a measure of the heat transfer due to phase change of a liquid during boiling. It is related to the heat flux that is a heat flow per unit area, and the thermodynamic driving force for the heat flow, i.e. a temperature difference.

An interesting advantage of using nanofluids for heat transfer applications is the ability to alter their properties. That is, the thermal conductivity and surface wettability, for example, can be adjusted by varying the particle concentration in the base fluid, and hence allowing nanofluids to be used for a variety of different applications. However, it is also important to note that addition of nanoparticles to a base fluid also changes the viscosity, density and even the effective specific heat; these properties also have a direct effect on the heat transfer effectiveness.

An enhancement of the CHF offers the potential for major performance improvement in many practical applications that use nucleate boiling as their primary heat transfer mode. To implement such heat transfer enhancements in the various applications previously listed, it is of paramount importance to better comprehend the fundamental BHT characteristics of nanofluids and the mechanisms that are at play in both convective and pool boiling regimes.

### Nanofluids enhancement on boiling

**Figure 1:**
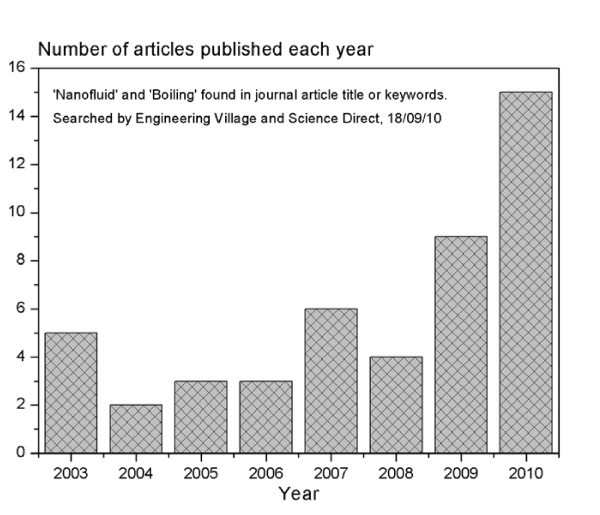
**Bar chart to illustrate the increasing trend in journal articles dedicated to nanofluid boiling in the last seven years**.

There are several review articles concerning nanofluids; some on their potential benefits on heat-transfer applications [5-11] and also some on their thermal conductivity enhancement [3,12]. The use of nanofluids for boiling enhancement is a promising area that is currently being explored by many researchers for pool boiling applications [4,13-16], and more recently, albeit to a lesser extent, in convective boiling applications [17,18]. Figure 1 shows the rapid growth in nanofluid boiling research in recent years. The articles shown in the bar chart of Figure 1 are those that have been published in journals between 2003 and 2010; before 2003, there were no published journal articles found using both keywords 'nanofluid' and 'boiling'. (The authors would like to point out that there have been conference articles concerning 'nanofluids' and 'boiling', but only published journal articles have been considered in Figure 1). There is a sharp increase in nanofluid boiling research in recent years; this is most likely due to the reported enhanced thermal conductivity of nanofluids, and the relatively large gap in the knowledge that exists, concerning the mechanisms involved in nanofluid boiling enhancement.

This review article has tried to incorporate all dominant pool boiling and convective boiling articles using nanofluids to date. A summary of the main convective and pool nanofluid boiling studies has been provided in Table 1. It is hoped that this article provides a concise and fair account of the advantages and of the limitations of nanofluids in respect of their boiling performance and application.

**Table 1:** Summary of the main convective and pool boiling nanofluid journal articles in the last seven years

Author names [reference]	Year	Type of boiling	Heater type	Nanofluid	Relevant information
Faulkner et al. [19]	2003	Convective	-	Ceramic nanoparticles in water	Parallel microchannel heat sink Limited improvement in overall heat transfer rate with nanofluid
Lee and Mudawar [18]	2007	Convective	-	Al_2_O_3 _nanoparticles in water	Microchannel (copper) cooling operations Single-phase, laminar flow → CHF enhancement Two-phase flow → nanoparticle agglomerates at channel exit, catastrophic failure
Peng et al. [20]	2009a	Convective	-	CuO nanoparticles in R-113	Flow boiling inside copper tube BHT enhancement (up to 30%) Enhancement caused by reduction of boundary layer height, due to disturbance of nanoparticles and formation of molecular adsorption layer on nanoparticle surface
Peng et al. [21]	2009b	Convective	-	CuO nanoparticles in R-113	Flow boiling inside copper tube Frictional pressure drop larger (up to 21%) than pure R-113, and increases with nanoparticle concentration
Boudouh et al. [22]	2010	Convective	-	Copper nanoparticles in water	50 parallel minichannels of *d*_h _= 800 μm Local BHT increases with nanoparticle concentration Higher Δ*P *and lower *T*_surface _with nanofluid compared to pure water at same mass flux Cu-water nanofluid suitable for microchannel cooling
Kim et al. [23]	2010	Convective	-	Al_2_O_3_, ZnO, and Diamond nanoparticles in water	CHF enhancement (up to 53%), increased with mass flux and nanoparticle concentration BHT small enhancement at low heat flux Nanoparticle deposition on heater → CHF enhancement
Kim et al. [24]	2010	Convective	-	Al_2_O_3 _nanoparticles in water	CHF enhancement (up to 70%) at low nanoparticle concentration (<0.01 vol.%) Nanoparticle deposition on heater surface → wettability increased
Henderson et al. [25]	2010	Convective	-	SiO_2 _nanoparticles in R-134a and CuO nanoparticles in R-134a/polyolester oil	BHT deterioration by 55% compared to pure R-134a Nanoparticle deposition on copper tube walls
Ahn et al. [17]	2010	Convective and pool	Cu plate	Al_2_O_3 _nanoparticles in water	CHF enhancement for Pool and Convective boiling Enhancement due to nanoparticle deposition on heater surface → wettability increased
You et al. [4]	2003	Pool	Cu plate	Al_2_O_3 _nanoparticles in water	CHF enhancement (up to 200%) BHT unchanged Enhancement not related to increased thermal conductivity of nanofluids
Witharana [26]	2003	Pool	Cu plate	Au nanoparticles in water	BHT increase (between 11 and 21%) at low nanoparticle concentrations (0.001 wt%) Increasing particle concentration, BHT enhancement increased
Das et al. [13]	2003a	Pool	Cylinder cartridge heater	Al_2_O_3 _nanoparticles in water	BHT degradation & wall superheat increase with increasing nanoparticle concentration Limited application for boiling of nanofluids Nanoparticle deposition on heater surface
Das et al. [27]	2003b	Pool	Stainless steel tubes	Al_2_O_3 _nanoparticles in water	BHT degradation & increase in wall superheat with increasing nanoparticle concentration Boiling performance strongly dependent on tube diameter BHT degradation less for narrow channels than for larger channels at high heat flux
Vassallo et al. [28]	2004	Pool	NiCr wire	SiO_2 _nanoparticles in water	CHF enhancement (up to 60%) No change in BHT
Wen and Ding [29]	2005	Pool	Stainless steel plate	Al_2_O_3 _nanoparticles in water	CHF enhancement (up to 40%) Nanoparticle deposition on heater surface
Bang and Chang [30]	2005	Pool	Stainless steel plate	Al_2_O_3 _nanoparticles in water	CHF enhancement (up to 50%) BHT degradation Nanoparticle deposit on heater surface, porous layer formed → wettability increased
Milanova and Kumar [31]	2005	Pool	NiCr wire	SiO_2 _nanoparticles in water (also in salts and strong electrolyte solution)	CHF enhancement three times greater than with pure water Nanofluids in salts minimise potential increase in heat transfer due to clustering Nanofluids in a strong electrolyte, higher CHF obtained than in buffer solutions due to difference in surface area
Kim et al. [32]	2006	Pool	Stainless steel plate	Al_2_O_3_, ZrO_2 _and SiO_2 _nanoparticles in water	Nanoparticle deposition on heater surface Irregular porous structure formed Increased wettability → CHF enhancement
Kim et al. [33]	2006a	Pool	NiCr wire	TiO_2 _nanoparticles in water	CHF enhancement (up to 200%)
Kim et al. [34]	2006b	Pool	NiCr and Ti wires	Al_2_O_3 _and TiO_2 _nanoparticles in water	CHF enhancement Nanoparticle deposition on heated wire CHF of pure water measured using a nanoparticle-coated heater Nanoparticle deposition on heater → CHF enhancement
Chopkar et al. [35]	2007	Pool	Cu surface	ZrO_2 _nanoparticles in water	BHT unchanged Surfactants added to nanofluid as a stabiliser Boiling renders heater surface smoother
Kim et al. [36]	2007	Pool	Stainless steel wire	Al_2_O_3_, ZrO_2 _and SiO_2 _nanoparticles in water	CHF enhancement (up to 80%) at low concentrations (<0.1 vol.%) Nanoparticle deposition on heater surface → porous layer, wettability increased BHT deterioration
Kim et al. [37]	2007	Pool	NiCr wire	Al_2_O_3 _and TiO_2 _nanoparticles in water	CHF enhancement (up to 100%) Nanoparticle deposition on heater surface Increased wettability → CHF enhancement
Park and Jung [38]	2007	Pool	Stainless steel tube	Carbon nanotubes (CNT) in water and R-22	CNTs increase BHT (up to 29%) for both base fluids No surface fouling observed with CNTs
Ding et al. [39]	2007	Pool	Stainless steel plate	Al_2_O_3 _and TiO_2 _nanoparticles in water	BHT enhancement for both TiO_2 _and Al_2_O_3_ BHT enhancement increases with nanoparticle concentration, and enhancement is more sensitive for TiO_2 _than Al_2_O_3 _→ nanoparticle properties affect BHT
Coursey and Kim [40]	2008	Pool	Cu and CuO plates, and glass, and gold coated plates	Al_2_O_3 _nanoparticles in ethanol and also in water	Strong relationship between boiling performance and fluid/surface combination and particle concentration CHF enhancement (up to 37% for poor wetting system) CHF enhancement mechanism is ability of fluid to improve surface wettability Surface treatment alone resulted in similar CHF enhancement as nanofluids, but at 20°C lower wall superheat
Milanova and Kumar [41]	2008	Pool	NiCr wire	SiO_2 _nanoparticles in water	CHF enhancement 50% with no nanoparticle deposition on wire CHF enhancement three times greater with nanoparticle deposition
Liu and Liao [42]	2008	Pool	Cu plate	CuO and SiO_2 _nanoparticles in water and (C_2_H_5_OH)	BHT degradation as compared to pure base fluids CHF enhancement Nanoparticle deposition on heater surface → wettability increased
Trisaksri and Wongwises [43]	2009	Pool	Cu cylindrical tube	TiO_2 _nanoparticles in R-141b	BHT deteriorated with an increase in nanoparticle concentration At low concentrations (0.01 vol%), no effect on BHT
Golubovic et al. [44]	2009	Pool	NiCr wire	Al_2_O_3 _and Bismuth oxide (Bi_2_O_3_) nanoparticles in water	CHF enhancement (up to 50% for Al_2_O_3 _and 33% for Bi_2_O_3_) CHF increases with nanoparticle concentration, until a certain value of heat flux Average particle size has negligible effect on CHF Nanoparticle material effects CHF Nanoparticle deposition on heater surface → wettability increased
Kim et al. [45]	2010	Pool	NiCr wire	Al_2_O_3 _and TiO_2 _nanoparticles in water	CHF enhancement, with large wall superheat Nanoparticle deposition on heater surface, surface modification results in same CHF enhancement in pure water as for nanofluids Nanoparticle layer increases stability of evaporating microlayer under bubble
Soltani et al. [46]	2010	Pool	Stainless steel cartridge heater	Al_2_O_3 _nanoparticles in CMC solution (carboxy methyl cellulose)	BHT degradation, more pronounced at higher CMC concentrations BHT enhanced with nanoparticles and CMC solution, and BHT increases with nanoparticle concentration (up to 25%)
Liu et al. [47]	2010	Pool	Cu plate	Carbon nanotubes (CNTs) in water	CHF and BHT enhancement CNT concentration has strong influence on both BHT and CHF enhancement, an optimal mass concentration of CNTs exists Decrease in pressure, increase in CHF and BHT enhancement CNT porous layer deposited on heater surface after boiling
Kwark et al. [15]	2010	Pool	Cu plate	Al_2_O_3_, CuO and diamond nanoparticles in water	CHF enhancement CHF increases with nanoparticle concentration, until a certain heat flux CHF enhancement potential decreases with increasing system pressure BHT coefficient unchanged After repeated testing, CHF remains unchanged, but BHT degrades 3 nanofluids exhibit same performance Nanoparticle deposit on heater surface Investigated mechanisms behind nanoparticle adhesion and surface deposit
Suriyawong and Wongwises [48]	2010	Pool	Cu and Al plates	TiO_2 _nanoparticles in water	2 surface roughness (0.2 and 4 μm) 4 μm roughness gives higher BHT than 0.2 μm roughness *Copper surfaces* At low nanoparticle concentrations BHT increased (15% at 0.2 μm, and 4% at 4 μm roughness) *Aluminium surfaces* BHT degraded for all nanoparticle concentrations and surface roughness

### Convective flow boiling

Research in convective flow boiling of nanofluids has become more popular in the past two years, perhaps because of the recent demand for high-heat flux cooling of microelectronics components and other compact cooling processes. An experimental study was conducted by Lee and Mudawar [18] to explore the benefits of using alumina (Al_2_O_3_) nanoparticles in a water base fluid for microchannel-cooling applications. They found enhancement of the heat-transfer coefficient for single-phase laminar flow; however, in the two-phase regime, the nanofluids caused surface deposition in the microchannels, and large clusters, agglomerates of nanoparticles, were formed. This clogging problem is a serious issue if nanofluids are to be incorporated in microchannel cooling of microelectronics components, where any temperature excursions can result in temperature hot spots and possible thermal failure of the device.

**Figure 2:**
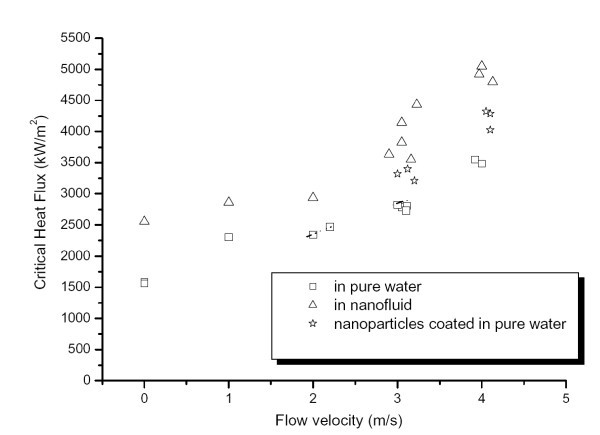
**Comparisons of CHF values for pure water and nanofluid on the clean surface, and pure water on a nanoparticle-coated surface **[17].

As stated previously in the *Introduction*, only low volume concentrations of nanoparticles are required to significantly alter the thermal properties of the base fluids. Ahn et al. [17] investigated aqueous nanofluids with a 0.01% concentration of alumina nanoparticles; CHF was distinctly enhanced under forced convective flow conditions compared to that in pure water; see Figure 2. They conducted experiments with varying flow velocities, starting from 0 m/s (effectively pool boiling) up to 4 m/s. A CHF enhancement of 50% was found at 0 m/s, which is consistent with pool boiling CHF enhancement found by previous researchers [30,45]. After the boiling experiments, these authors used a scanning electron microscope (SEM) to examine the heater surfaces, and the contact angle was also measured. They determined that the enhancement was mainly due to nanoparticle deposition on the heater surface during vigorous boiling. This deposition caused the contact angle to decrease from 65° to about 12°, illustrating an evident enhancement in the wettability of the heater surface. The experiments performed by Ahn et al. illustrated that nanofluids caused significant CHF enhancements for both pool boiling and convective flow boiling conditions. Figure 2 shows the comparison between the CHF values for water boiling on both a clean surface and on a nanoparticle-fouled surface. Flow boiling CHF enhancement in nanofluids is strongly related to the surface wettability, which is similar to the pool boiling CHF enhancement as will be discussed in the following section on 'Pool boiling'.

Another investigation by Kim et al. [23] also resulted in a similar nanoparticle deposition on the heater surface after nanofluid boiling. Kim et al. [23] investigated the subcooled flow boiling using dilute alumina, zinc oxide and diamond water-based nanofluids. They measured both the CHF and the heat transfer coefficient during their flow boiling experiments. CHF enhancement was found to increase with both mass flux and nanoparticle concentration for all nanoparticle materials; an increase as great as 53% was observed for CHF. The experimental data obtained for the heat transfer coefficient showed little enhancement for the nanofluids at low heat fluxes; a slight enhancement was seen at higher heat fluxes. They also arrived at the same theory as Ahn et al. [17]; that is, the nanoparticle deposition on the heater is one of the main contributors to the CHF enhancement. In relation to how this nanoparticle deposit can affect the heat transfer coefficient, they came to two conclusions: firstly, that the deposit changes the number of micro-cavities on the surface, and secondly that the surface wettability is also changed. They measured the number of micro-cavities on the surface and the contact angle of the fluid on the surface, and hence obtained an estimation of the nucleation site density at the heater surface. However, whether the nucleation site density was enhanced or found to deteriorate, the heat transfer coefficient remained largely unchanged as that obtained for pure water. They concluded from this that there must be other mechanisms offsetting the effect of nucleation site density enhancement, possibly changes in the bubble departure diameter and/or bubble departure frequency.

Again, Kim et al. [24] noticed a nanoparticle deposition on the heater surface after nanofluid flow boiling, and considered this to be the main cause behind the CHF enhancement that they observed. They found a CHF enhancement of up to 70%, with a nanoparticle content of less than 0.01% by volume of alumina in water. This again shows that only a small nanoparticle concentration is required to obtain rather dramatic CHF enhancements during flow boiling of nanofluids.

Further experimental data need to be obtained on flow boiling of nanofluids, so as to have a more substantial database, and a better understanding on nanofluid flow boiling mechanisms. In contrast, there is a much greater number of nanofluid pool boiling experiments available in the literature, which are discussed in the following section on 'Pool boiling'.

### Pool boiling

Pool-boiling experiments with water-based nanofluids containing Al_2_O_3_, ZrO_2 _and SiO_2 _nanoparticles were conducted by Kim et al. [32]. Again, nanoparticle deposition was observed on the heater surface soon after nanofluid boiling was initiated; an irregular porous structure was formed at the surface. This is very similar as to the one that was observed during the convective flow boiling of nanofluids presented in the previous section. Kim et al. [32] investigated this surface deposition further and noted an enhancement in wettability. They analysed the modified Young's equation and came to the conclusion that wettability enhancement is caused by two combined effects; the first effect they thought to be an increase in adhesion tension; and the second, an increase in the surface roughness. Activation of micro-cavities on the heater surface is inhibited by the nanoparticle deposition (since there is a decrease of contact angle), which leads to a decrease in bubble nucleation in nanofluids. The surface wettability affects the CHF; CHF occurs when dry patches (hot spots) develop on the heater surface at high heat fluxes; these dry spots can be rewetted or can irreversibly overheat, causing CHF. Therefore, an increase in surface wettability promotes dry-spot rewetting, thus delaying CHF.

**Figure 3:**
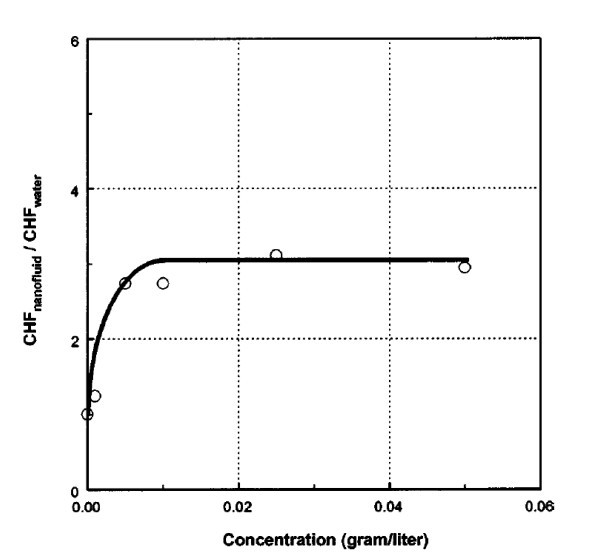
**Graph illustrating CHF_nanofluids_/CHF_water _at different concentrations (g/l) of nanoparticles **[4].

As presented previously in the section on 'Convective flow boiling', the addition of just a small volume concentration of nanoparticles can provide a significant CHF enhancement, and the same has been achieved during pool boiling of nanofluids as observed by You et al. [4] in 2003. You et al. measured the CHF in pool boiling using a flat, square copper heater submerged with nanofluids at a sub-atmospheric pressure of 2.89 psia. It should be noted here that in the literature, the pressure has been shown to have a great impact on the BHT and CHF enhancement, with both increasing significantly with a decrease in the system pressure [47]. The graph in Figure 3 evidences the effect of nanoparticle concentration on the CHF compared to a pure water case. You et al. noted that a 200% CHF increase was measured for a nanofluid containing just 0.005 g/l (approx. 10^-4 ^vol.%) of alumina nanoparticles.

Nanofluids were also found by Kim et al. [45], to significantly enhance the CHF, creating a large wall superheat during pool boiling of water-based nanofluids with 0.01% alumina and titanium nanoparticles. Once again, nanoparticle deposition was observed on the heater surface after vigorous nanofluid boiling. The enhancement of the CHF was found to be of the same magnitude when both nanofluids and pure water were later boiled on the already nanoparticle-fouled heater surface. This implies that the surface modification due to the deposition is the reason behind the CHF enhancement, and that perhaps the working fluid has little effect on the CHF, once the heater surface has already been nanoparticle-fouled. They went on to postulate that the nanoparticle layer increases the stability of the evaporating microlayer underneath a growing bubble on a heated surface, and thus irreversible growth of a hot spot is inhibited, resulting in CHF enhancement when boiling nanofluids.

**Figure 4:**
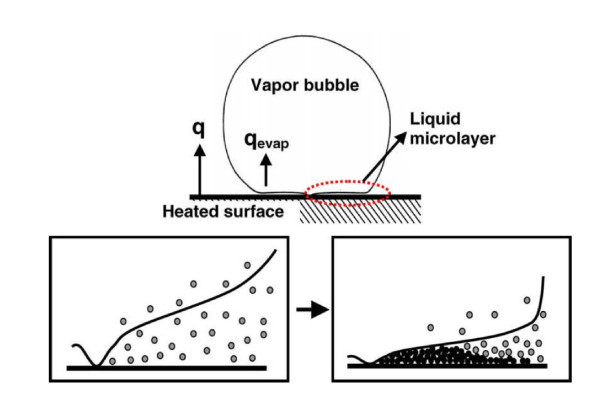
**Mechanism of nanoparticle deposition during the boiling process (micro-layer evaporation) **[15].

Further nanoparticle deposition was observed by Bang and Chang [30], who also measured a CHF enhancement of 50%, with alumina-water nanofluids on a stainless steel plate. They determined that the nanoparticle deposition on the heater after boiling was a porous layer that led to increased surface wettability. However, they also noted a deterioration in the BHT coefficient, which could have been an unfortunate result of the nanoparticle-fouled surface. Das et al. [13] also observed nanoparticle deposition on the heater surface after boiling. They too noted an increase in wall superheat with increasing nanoparticle concentration, and again degradation in the BHT with the alumina-water nanofluid that they investigated. Kwark et al. [15] postulated that the decrease in the BHT coefficient with increased nanoparticle concentration, which they observed, can be attributed to the corresponding thicker coating created, which offers increased thermal resistance. CHF, on the other hand, is not dictated by the thickness of the nanoparticle coating, but by the increased wettability that the nanoparticle deposit provides at the heater surface [36]. They concluded that there is an optimal nanofluid concentration, at which point the CHF enhancement is at a maximum, and without any degradation of the BHT coefficient. They found the optimal concentration to be about 0.025 g/l, and this is also consistent with data found in other studies [4]. They also demonstrated how the nanofluid boiling performance shows transient-like behaviour dependent on both heat flux and experiment duration, that is prolonging the nanofluid experiments adversely affects the BHT coefficient. Kwark et al. [15] also investigated possible mechanisms behind the deposition and adhesion of nanoparticles to the heater surface during boiling of nanofluids. Figure 4 illustrates the mechanism as proposed by Kwark et al. [15], where it is the boiling itself that appears to be the mechanism responsible for the nanoparticle coating formation. This is also consistent with Kim et al. [36], who postulated that nanoparticles are deposited on the heater surface during nanofluid boiling, hence creating a nanoparticle coating. They assumed that the nanoparticle coating was formed by nucleated vapour bubbles growing at the heater surface and the evaporating liquid that is left behind, inducing a concentrated micro-layer of nanoparticles at the bubble base.

**Figure 5:**
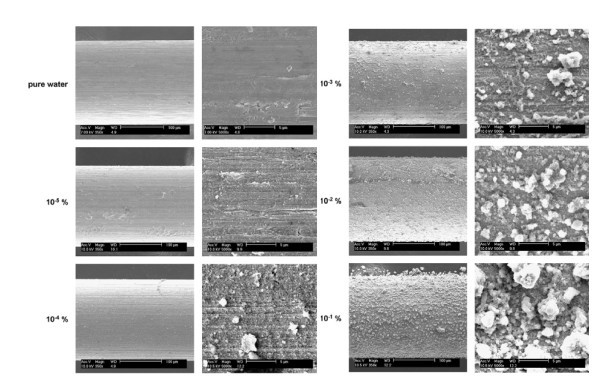
**TiO_2 _nanoparticle-coated NiCr wire after pool boiling CHF experiment of nanofluids with different particle volume concentrations **[34].

CHF enhancement in nanofluids has been widely observed by almost all researchers in convective boiling [17,23,24] and in pool boiling [4,15,17,28-34,36,37,40-42,44,45,47]. On the other hand, the BHT coefficient database is fairly inconsistent, and the data are rather scattered. Some researchers report no change of heat transfer in the nucleate boiling regime, some report heat transfer deterioration, and others heat transfer enhancement. Several studies (Kim et al. [36], Coursey and Kim [40], Kim et al. [34], Ahn et al. [17], Kim et al. [32], to name but a few) have attributed the CHF enhancement seen during both pool and convective boilings of nanofluids to the improved wettability at the heater surface after the deposition of a nanoparticle layer. Figure 5 clearly shows the nanoparticle deposit left on a NiCr wire after pool boiling of TiO_2 _nanoparticles, taken from Kim et al. [34].

The roughness of the nanoparticle-fouled surface is significantly greater than that of the clean surface, due to the nature of the peak-and-valley structure of the deposit. This surface roughness can affect the vapour bubble growth because of the distribution and activation of the nucleation sites.

Kwark et al. [15] performed two tests to investigate the effect of nano-coated surfaces on pool boiling performance. They used a clean heater with alumina (Al_2_O_3_) in water nanofluid, and also a nanoparticle-coated heater (this heater had been coated in a previous nanofluid boiling experiment) with pure water. Effectively, the first test built up the nanoparticle coating on the heater surface, and the second test investigated the effect of this coating on the boiling performance in pure water. They found that when the nano-coated heaters were tested in pure water, boiling on the surface may detach some of the nanocoating from the heater surface. However, the overall results showed that pure water with a pre-coated-nanoparticle heated surface provided the same CHF enhancement as nanofluids with the same nanoparticle-pre-coated heated surface, thus demonstrating that it is the surface coating and the enhanced wettability that cause the CHF enhancement that they observed, and not the suspended nanoparticles in the fluid (the nanofluid).

**Figure 6:**
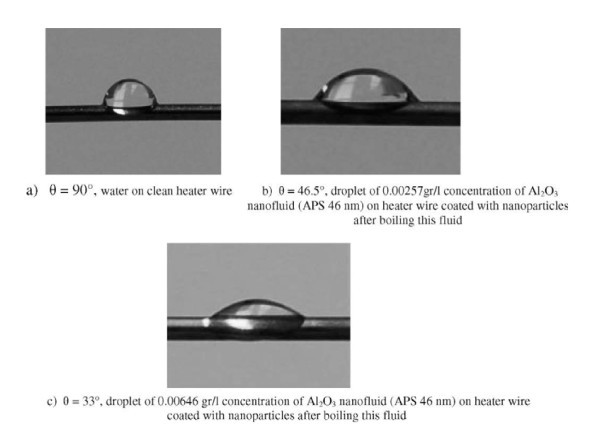
**Water and Al_2_O_3 _nanoparticle drops of different particle concentrations on heater surfaces boiled in corresponding nanoparticle concentration nanofluid **[44]. **(a) **θ = 90°, water on clean heater wire; **(b) **θ = 46.5°, droplet of 0.00257 g/l concentration of Al_2_O_3 _nanofluid (APS 46 nm) on heater wire coated with nanoparticles after boiling this fluid; **(c) **θ = 33°, droplet of 0.00646 g/l concentration of Al_2_O_3 _nanofluid (APS 46 nm) on heater wire coated with nanoparticles after boiling this fluid.

Nanofluid use in BHT has been shown in most cases to contribute to CHF enhancement. Research on surface characteristics indicates that deposition of nanoparticles on the heating surface is one of the main causes behind the CHF enhancement. Surface wettability, liquid spreadability and morphology are some of the heater surface properties altered by the nanoparticle deposition. Figure 6 illustrates how the contact angle drastically changes, dependent on whether the heated surface has been exposed to nanofluid boiling or not. The wettability also changes depending on the nanoparticle concentration in the base fluid, with a two-fold increase in the concentration of Al_2_O_3 _nanoparticles in water decreasing the contact angle from 46.5° to 33°.

Particle image velocimetry (PIV) has been used to help better comprehend the effects of nanofluids upon boiling. Dominguez-Ontiveros et al. [49] investigated Al_2_O_3 _nanoparticles in water, and visually observed their effect on nucleate boiling. They noted a change in the hydrodynamic behaviour of bubbles with the addition of nanoparticles to the pure water. Fluid velocities were depressed with nanofluids relative to the pure water case, and they also observed an increase in fluid circulation because of the nanoparticles. A relationship between wall temperature and nanoparticle concentration was found, and the complexity of the nanofluid pool boiling was highlighted. Further research of this nature, that is, the use of high-speed imaging, infrared thermography, PIV techniques, are required to fully comprehend the mechanisms of nanofluid boiling and the role of nanofluids on the enhancement phenomena observed by researchers.

## Discussion-advantages and disadvantages with nanofluids

Boiling with nanofluids enables certain properties to be adjusted by varying the nanoparticle concentration or nanoparticle material, such as the thermal conductivity of the working fluid and the surface wettability of the heater surface. The benefit of less pumping power required for the same heat transfer, compared to just using the base liquid, is also applicable. Nanofluid boiling also results in a build-up of a porous layer of nanoparticles on the heater surface. This layer has been shown to significantly improve the surface wettability; see Figure 6 where the measured changes in the static contact angle on the nanofluid-boiled surfaces compared with the pure-water-boiled surfaces are shown. It is hypothesised that this surface wettability improvement may be responsible for the CHF enhancement observed by almost all of the researchers so far. However, this nanoparticle layer is also considered by some researchers to be also responsible for the deterioration found in the BHT coefficient. Since the nanoparticle deposit creates a resistance in the heat transfer from the heater surface to the fluid, caused by a decrease in the contact angle, and/or produces a reduction in the nucleation site density. The heat transfer mechanisms responsible for the CHF and BHT enhancements and/or deteriorations have not been fully comprehended.

An article by Keblinski et al. [50] is a good overview of enhanced heat conduction in nanofluids, and the possible mechanisms involved. Several mechanisms for the enhancement of thermal conductivity are presented in their article such as Brownian motion of the particles, molecular-level layering of the liquid at the liquid-particle interface and the clustering effect of nanoparticles leading to direct solid-solid paths. Boiling enhancement in nanofluids is thought to be due to several mechanisms: firstly an enhancement via nanoparticle interactions with bubbles [46]; secondly, an improvement in the thermal conductivity at the heater surface due to the accumulation of highly conductive nanoparticles forming a porous deposit there [32]. Several researchers have noticed this nano-deposition at the heater surface, which can alter the surface area, the surface wettability and the bubble nucleation. Conversely, the nanoparticles gathering at the heater surface as a deposit results in a decrease in the number of nanoparticles available to interact with bubbles. Also the nanoparticle deposit at the heater can result in a loss of nucleation sites at the surface, since the nanoparticles may fill the micro-cavities, resulting in a loss of boiling performance [13,23,30,32,51]. The nucleation site density, bubble departure diameter and bubble frequency are all affected by nanofluid boiling. It has been found by several researchers [4,32] that bubble diameters increase during boiling with nanofluids, but the nucleation site density decreases with the addition of nanoparticles to the base fluid. Further studies focusing on bubble dynamics and bubble parameters will provide valuable insight into the mechanisms by which nanoparticles affect the heat transfer coefficient.

The research in the literature points to the fact that there is indeed a critical limit for the concentration of nanoparticles in a base fluid that will provide both CHF and BHT enhancements through particle interaction and nanoparticle deposition at the heater surface, but before too many boiling cavities are filled with nanoparticles. Previously illustrated in Figure 3 were the experimental data obtained by You et al. [4], which clearly indicated that there was a certain concentration (<0.01 g/l) after which no further CHF enhancement was found. The same conclusion was identified by Liu et al. [47], who found that an optimal carbon nanotubes mass concentration existed, which provided a corresponding maximum heat transfer enhancement in their experiments.

Formulating stable nanoparticle-in-liquid suspensions (nanofluids) is difficult, and so too is the control of their properties such as thermal conductivity, viscosity and wettability for heat transfer applications. There are some concerns over the dispersion stability of nanofluids [6,25,52,53] and of a particle migration effect occurring [29]. Certain approaches in preparation of nanofluids can lead to instability problems caused by particle agglomeration in the base fluid. Several researchers have experienced poor stability of nanofluids with sedimentation characteristics occurring. The addition of surfactants (or stabilisers) to nanofluids during the formulation process has been shown to effectively disperse nanoparticles in the base fluids. However, the addition of a surfactant can greatly change the properties of the nanofluid. For example, the surface tension, viscosity and wettability can all be altered, and so the properties of the nanofluid should include the effect of addition not only of the nanoparticles to the base fluid, but also of the surfactant. This could be a reason for the scattering of data found in Table 2 as it is difficult to differentiate whether it is the nanoparticles or the surfactant, which have altered the thermal properties of the base fluid, and it was also not clear in some articles in the literature if a stabiliser or surfactant had been added to the nanofluid. Factors, such as time, temperature, concentration, particle type, dispersion medium and pH, all play important parts in the dispersion stability, with poor dispersion of nanoparticles in the base fluid possibly resulting in poor heat transfer enhancement. It is also essential to have a uniformly dispersed nanofluid when obtaining heat transfer data; otherwise the data will not necessarily be easy to reproduce. It could hence be beneficial to validate the dispersion of nanoparticles in their base fluids with the use of scattering techniques, hence providing a characterisation of the particle distribution.

**Table 2:** Summary of the effect of nanofluids on the BHT coefficient and on the CHF.

Author names and [reference]	Year	BHT effect and (nanoparticle type)	CHF effect
Witharana [26]	2003	Enhancement between 11 and 21% (Au, SiO_2 _on Cu surface)	Enhancement
Wen and Ding [29]	2005	Enhancement up to 40% (Al_2_O_3_) Enhancement up to 50% (TiO_2_)	Enhancement
Ding et al. [39]	2007	Enhancement (Al_2_O_3_, TiO_2 _on S/S plate)	-
Park and Jung [38]	2007	Enhancement up to 29% (carbon nanotubes on S/S tube)	-
Peng et al. [20]	2009	Enhancement up to 30% (CuO/R-113)	-
Boudouh et al. [22]	2010	Enhancement (Cu)	-
Kim et al. [23]	2010	Small enhancement (Al_2_O_3_, Zinc oxide and diamond)	Enhancement, up to 53%
Soltani et al. [46]	2010	Enhancement up to 25% (Al_2_O_3_/water and CMC on S/S heater)	-
Liu et al. [47]	2010	Enhancement (carbon nanotubes on Cu plate)	Enhancement
Suiyawong and Wongwises [48]	2010	Enhancement up to 15% (TiO_2 _on Cu surface)	-
Das et al. [13,27]	2003a, b	Deterioration between 10 and 40% (Al_2_O_3 _on S/S tubes)	-
Bang and Chang [30]	2005	Deterioration by approximately 20% (Al_2_O_3 _on S/S plate)	Enhancement, up to 50%
Kim et al. [36]	2007	Deterioration (Al_2_O_3_, ZrO_2_, SiO_2 _on S/S wire)	Enhancement, up to 80%
Liu and Liao [42]	2008	Deterioration (CuO, SiO_2 _in water and alcohol on Cu plate)	Enhancement
Trisaksri and Wongwises [43]	2009	Deterioration (TiO_2_/R-141b on Cu surface)	-
Suiyawong and Wongwises [48]	2010	Deterioration (TiO_2 _on Al surface)	-
Henderson et al. [25]	2010	Deterioration by 55% (SiO_2_/R-134a)	-
You et al. [4]	2003	Unchanged (Al_2_O_3 _on Cu surface)	Enhancement, up to 200%
Vassallo et al. [28]	2004	Unchanged (SiO_2 _on NiCr wire)	Enhancement, up to 60%
Chopkar et al. [35]	2007	Unchanged (ZrO_2 _on Cu surface)	-
Kwark et al. [15]	2010	Unchanged (Al_2_O_3_, CuO and diamond on Cu plate)	Enhancement

All nanoparticles have water as the base fluid, unless otherwise stated.

The scatter of nanofluid boiling data, as shown in Table 2 could hence be due to the nature of the nanofluids used and to what extent the nanoparticles remained suspended in the base fluid, as discussed previously. It has already been shown in the literature that during two-phase cooling in a microchannel [18], nanoparticles can cause catastrophic failure by depositing into large clusters near the channel exit due to localised evaporation once boiling commences. There is some uncertainty over whether degradation over time occurs on the enhancement effect of nanofluids and nanocoatings on the BHT. Table 2 clearly illustrates the conflicting data existing in the literature on the effect of nanofluids on the BHT coefficient. However, it is almost conclusive that the presence of nanoparticles suspended in a base fluid does increase the critical heat flux of the boiling system.

**Figure 7:**
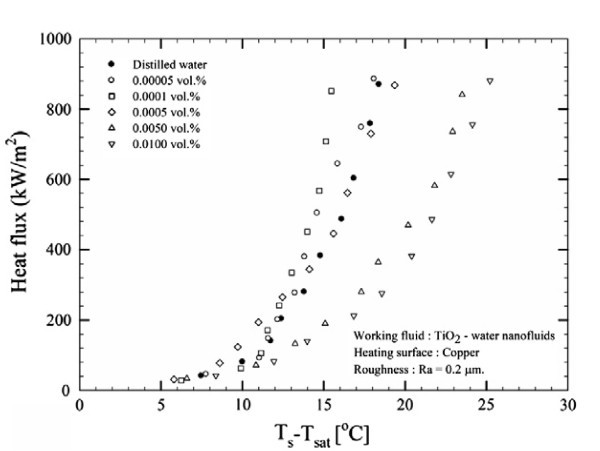
**Nucleate pool BHT of TiO_2_-water nanofluids for copper heating surface with roughness 0.2 μm at 1 atm **[48].

**Figure 8:**
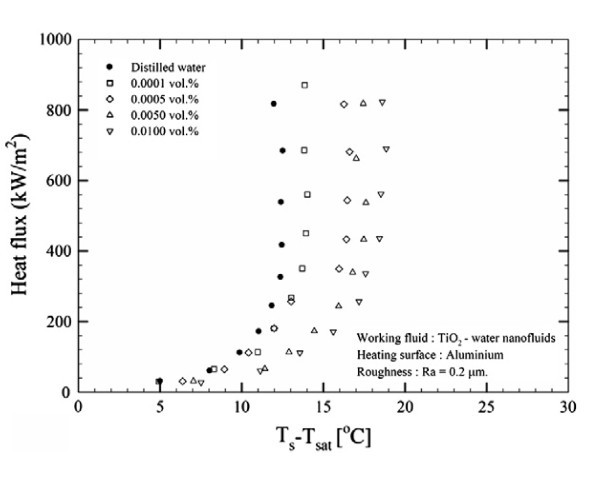
**Nucleate pool BHT of TiO_2_-water nanofluids for aluminium heating surface with roughness 0**.2 μm at 1 atm [48].

**Figure 9:**
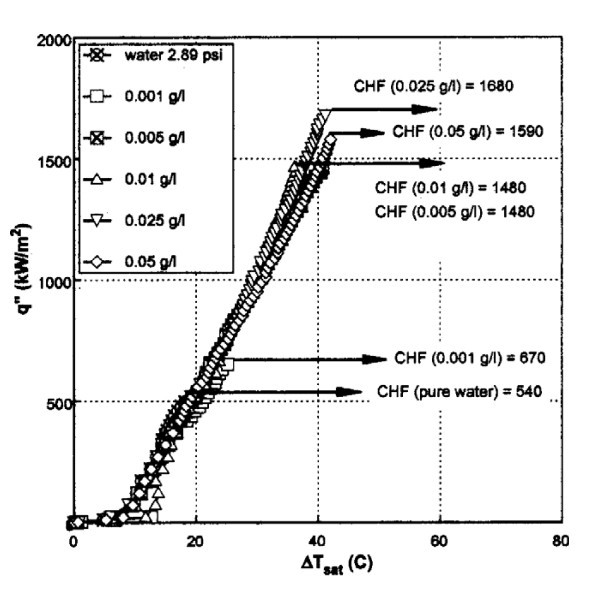
**Boiling curves at different concentration of Al_2_O_3_-water nanofluids during pool boiling **[4].

To better understand the use of the terms 'enhancement', deterioration' and 'unchanged' as used in Table 2 boiing heat transfer experimental data have been provided for each of these three terms, see Figures 7, 8 and 9.

Figures 7, 8 and 9 illustrate BHT 'enhancement, 'deterioration' and 'unchanged', respectively. It can be seen in Figure 7 that the BHT is enhanced with the TiO_2_-water nanofluid at the two smallest concentrations of 0.00005 and 0.0001 vol.%, as investigated by Suriyawong and Wongwises [48]. After 0.0001 vol.%, the BHT starts to deteriorate. It is interesting to note that Figure 8 shows experimental data from the same researchers [48], except that the TiO_2 _nanofluid was boiled on an aluminium surface as opposed to a copper surface as seen in the previous figure, Figure 7. The combination of the TiO_2 _nanofluid with the aluminium surface led to deterioration in the BHT for all the nanoparticle concentrations investigated. Finally, Figure 9 shows experimental data of You et al. [4], whose investigations of Al_2_0_3_-water nanofluids on copper surfaces showed no evident change in the BHT over that obtained for pure water. The results presented in the literature are inconsistent even for nanoparticles under similar experimental conditions.

## Conclusions

Nanofluids have been shown by nearly all researchers to enhance the CHF during boiling. However, there are conflicting experimental results regarding the effect that nanofluids have on the BHT coefficient, as shown in Table 2. Some researchers have shown that nanofluids provide an enhancement [20,29] on the BHT coefficient, others a deterioration [13,30], and some others no change at all [4,28]. Further systematic experimental study needs to be performed to understand the mechanisms behind BHT enhancement, and to comprehend why such contradictory data exist among researchers. The BHT coefficient is an important factor, particularly if nanofluid boiling is to be incorporated in the design of engineering systems, such as the cooling of nuclear reactors.

**Figure 10:**
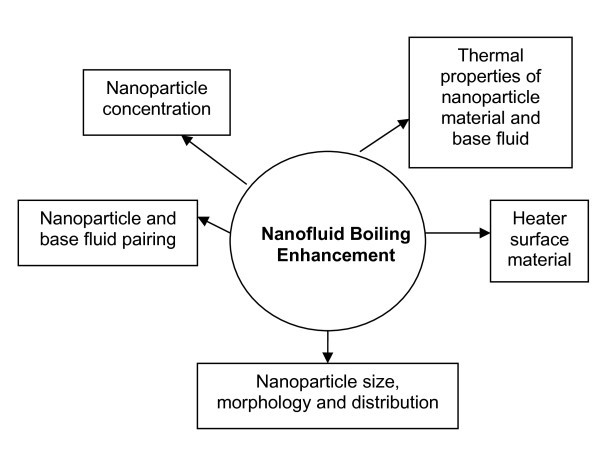
**Factors affecting nanofluid boiling enhancement**.

Figure 10 summarises pictorially the main factors affecting nanofluid boiling enhancement. It has been shown by researchers that there are several factors that individually or in combination can play an important role in the nanofluid boiling enhancement. For example, Suiyawong and Wongwises [48] noted an enhancement in the BHT of up to 15% when they investigated TiO_2 _pool boiling on copper surfaces, but a deterioration in the BHT when they boiled the same TiO_2 _nanofluid on an aluminium heater, see Figures 7 and 8.

Nanoparticle deposition on the heater surface has been observed by nearly all the researchers who have conducted nanofluid boiling, both pool and convective. This is thought to be the main reason behind the critical heat flux enhancement. This nanoparticle layer increases the surface roughness, the surface area, and the surface wettability. The mechanisms underlying this CHF enhancement have still not been clarified, and they remain under discussion and investigation.

Water has been the most commonly used working fluid with nanoparticles so far in the literature. It would be interesting to compare water-based nanofluids with heat-transfer data to be obtained for refrigerant-based nanofluids in the future. There exist already a few experimental studies using refrigerant-based nanofluids, e.g. Peng et al. [20,21], Henderson et al. [25] and Park and Jung [38]. More experimental data with varying base fluids is required. However, there have already been some reports [25] that water has the greatest aptitude to suspend non-coated nanoparticles, in comparison with other base fluids such as ammonia, hydrocarbons, HFCs and HCFCs.

Boiling performance is dependent on the combined effect of particle concentration, surface properties, and the nature of base fluid (i.e. if it is highly wetting), as indicated by Coursey and Kim [40]. If CHF enhancement is due to nanofluids reducing the contact angle, and due to improving wetting, then it might be advisable to simply provide surface treatment (nanocoatings) to the boiling surfaces as opposed to using nanofluids, since already surface oxidation alone has been shown to provide slightly higher heat transfer than nanofluids at a lower wall superheat by 20°C [40].

It has been shown in the literature that the use of nanofluids in boiling is a relevant and pertinent topic. There are many benefits of nanofluid boiling, particularly, in terms of increasing the CHF of the boiling system. However, further research is required before conclusive findings can be presented on the effect of nanofluid boiling on the BHT. It is also important to perform experiments over a long time period, to see if there are any time-dependent effects on the nanoparticle suspensions. Nanofluid boiling has resulted in most researchers finding a porous nanoparticle deposit on the heater surface after vigorous boiling. This deposit is considered by most researchers to be responsible for the CHF enhancement. If this is the case, then it could prove to be just as advantageous to simply pre-coat heater surfaces with nano-deposits instead of boiling with nanofluids, where possible flow passage blockages, particularly in convective flow boiling applications, could be prevented.

## Abbreviations

BHT: boiling heat transfer; CHF: critical heat flux; PIV: particle image velocimetry.
